# Glycemic index of different varieties of yam as influenced by boiling, frying and roasting

**DOI:** 10.1002/fsn3.2087

**Published:** 2020-12-25

**Authors:** Deborah Ampofo, Jacob K. Agbenorhevi, Caleb K. Firempong, Evelyn Adu‐Kwarteng

**Affiliations:** ^1^ Department of Biochemistry and Biotechnology Kwame Nkrumah University of Science and Technology Kumasi Ghana; ^2^ Department of Food Science and Technology Kwame Nkrumah University of Science and Technology Kumasi Ghana; ^3^ CSIR – Crops Research Institute Kumasi Ghana

**Keywords:** blood glucose response, carbohydrate, *Dioscorea alata*, *Dioscorea cayenesis*, *Dioscorea rotundata*, glycemic index

## Abstract

Yam is one of the commonly consumed carbohydrate staples. The objective of this work was to investigate the effect of boiling, roasting, and frying on the glycemic index (GI) of white yam (*Dioscorea rotundata*), yellow yam (*Dioscorea cayenesis*), and water yam (*Dioscorea alata*). Yam tubers were obtained (peeled, sliced, washed) deep fried in vegetable oil for 35–40 min and boiled in water for about 35–45 min. Sliced unpeeled tubers were also roasted at about 120°C for 40–45 min. The cooked yam samples were fed to 10 healthy subjects aged between 20–50 years. A glucometer was used to measure the blood glucose concentrations of the test individuals before consuming the yam diets and after the 15th, 30th, 45th, 60th, 90th, and 120th min of consumption. The average incremental area under the curves (IAUC) obtained from the recorded blood glucose concentrations were used to calculate the GI of various yam diets. The GI of the yam diets were found to be in the following increasing order: White‐yam‐boiled (44.26%) < Water‐yam‐boiled (50.12%) < White‐yam‐roasted (50.62%) < Water‐yam‐roasted (54.04%) < White‐yam‐fried (59.13%) < Yellow‐yam‐fried (65.08%) < Water‐yam‐fried (69.16%) < Yellow‐yam‐roasted (70.62%) < Yellow‐yam‐boiled (75.18%). White yam diets relatively had lower GI compared to yellow yam and water yam. Boiling was found to give generally lower GI in the white and water yams and could therefore be applied in the preparation of lower GI diets for diabetics.

## Graphical abstract

Glycemic index (GI) varied insignificantly among the yam types and cooking methods studied.

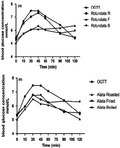

## 1 INTRODUCTION

Different carbohydrate foods when consumed have different impacts on blood glucose level and health (Englyst et al., 2007; Jenkins et al., 2000, 2002; Livesey et al., 2019). Carbohydrate foods that quickly breakdown into glucose after consumption are classified as a high glycemic index whereas those that slowly convert into glucose are also classified as low glycemic index. Foods with GI ranges from 0–55, 56–69, and 70–100 are classified as low, medium, and high GI foods, respectively (Eli‐Cophie et al., 2017; Yeboah et al., 2019). Factors that can influence the GI of a food include processing, variety, methods of cooking, ripeness, amylose‐to‐amylopectin ratio, dietary fiber, and storage time (Aston et al., 2008).

In most countries in the world, carbohydrates form the bulk of diet and are major societal staples (Mann et al., 2007). Carbohydrates also form the largest portion of the acceptable macronutrient distribution range and contribute 45%–70% of overall energy intake and usage (Elia & Cummings, 2007). Proper glycemic control among diabetics also requires careful selection of the carbohydrates portion of the food and this is the basis for the development of the carbohydrate exchange list to help with easier food selection. This exchange list comprises a group of carbohydrate foods containing the same amount of calories and with similar nutrients such that one could be interchanged for the other (Frost & Dornhorst, 2000; Hong et al., 2008). However, these exchange lists may not be so beneficial to controlling plasma glucose or hyperinsulinemia as foods in the same category may have different effects on blood glucose (Jenkins et al., 2000, 2002).

Yam is one of the commonly consumed carbohydrate staples in West Africa (Boateng et al., 2019). Ghanaian yam dishes including fried yam, boiled yam, *mpotompoto*, and yam *fufu* are cooked and accompanied with vegetable or protein‐based sauces and soups. The most common grown varieties of yam in Ghana and West Africa are *D. alata*, *D. rotundata*, and *D. cayensis* (Boateng et al., 2019).

Cooking of food improves the digestibility of the food and makes some nutrients more readily available. Different cooking processes have varying impacts on digestibility and as such glycemic index. Presence of other ingredients can also impact on glycemic index of a given carbohydrate food. Processing and cooking methods can bring variations in the glycemic index of one particular food. When carbohydrate foods are cooked, there is breakage of the cell wall and the starch within the food gelatinizes. This enhances accessibility of the starch to amylase leading to higher digestibility and a rise in glycemic index (Eli‐Cophie et al., 2017; Foster‐Powell et al., 2002; Yeboah et al., 2019). A study conducted by Bahado‐Singh et al. (2006) found that boiled foods such as potatoes, unripe bananas, and unripe plantain have lower glycemic indexes when boiled. This is due to the high presence of resistant starches in these foods. When cooling occurs after boiling resistant starch recrystallize, becomes closely bonded and inaccessible to amylase (Buyken & Kroke, 2005; Han et al., 2006). Nilsson et al. (2008) also found that boiling as a means of cooking was associated with low glycemic index.

Fat digestion is slow within the gut and its usage in cooking is hypothesized to lower glycemic index mainly by slowing the breakdown of starch (Ayodele & Godwin, 2010). Fernandes et al. (2005) also reported that glycemic index of fried potatoes was low because fat impeded the breakdown of starch. Fat might also decrease the glycemic index of food by increasing the quantity of resistant starch (Leeman et al., 2008). Increase in resistance starch slows the breakdown of the amylose–amylopectin bond and thereby lowers glycemic index. Additionally, fat and amylose may interact to form complexes that which may impede the hydrolysis of amylose (Leeman et al., 2008). Even though fried food are associated with low glycemic index, they are not encouraged as a means of controlling postprandial glycemia because they are high in fat and can lead to weight gain and dyslipidemia (Taveras et al., 2005). Farnetti et al. (2011) however found that food fried in virgin olive oil improved insulin response among obese patients with insulin resistance. In a study conducted by Bahado‐Singh et al. (2006), it was revealed that roasted potatoes had higher glycemic index compared to boiled potatoes. They hypothesized that the presence of intact skin around the roasted potatoes makes sugars more readily available leading to the increase in glycemic index. Also the loss of moisture during roasting reduces the formation of R3 resistant starches upon cooling and this occurrence may also increase glycemic index. Ayodele and Godwin (2010) in contrast found that roasted plantain which had a lower glycemic index compared to boiled and fried plantain. Björck et al. (2000) reported that the use of low heat during roasting preserves starch crystallinity which lower the rate of amylose hydrolysis and thus reduce glycemic index.

However, little has been done on the influence of variety and cooking methods on the glycemic index of yam which is commonly consumed in different forms in Ghana. The objective of this study, therefore, was to assess the influence of some cooking methods and variety on the glycemic indices of three local yam species.

## 2 MATERIALS AND METHODS

### 2.1 Study subjects

An approval was sought from the Committee on Human Research, Publication and Ethics of the Kwame Nkrumah University of Science and Technology School of Medical Sciences/Komfo Anokye Teaching Hospital, Kumasi, Ghana. A nutritional screening was carried out in School of dispensing optics, Oyoko, and about 150 people were screened. Based on the outcome ten (10) healthy individuals aged 20–50 years were selected with their consent. Thus these individuals were not morbidly obsessed and with no complain of ill health or any known metabolic disorder including insulin resistance.

Subjects were given an orientation before the start of the project. They were advised to sternly abstain from smoking or drinking of alcohol within the stipulated time of study and not to engage in any strenuous exercise or activity prior to the testing days. Data on the demographics and clinical history including history of diabetes, metabolic disorder or any cardiovascular diseases, time the last meal was taken the previous day, and the kind of food eaten by all participants were collected.

### 2.2 Anthropometric measurements of subjects

All subjects were made to undergo a 10–14 hr fast from the time of taking the last meal of the previous evening to the morning of testing. The heights of participants were measured with a stadiometer and their weights were also recorded using OMRON^®^ HBF 500 body composition monitor (Omron Corp.). All readings were replicated twice for each subject to ensure accuracy. The average heights and weights of each participant were used to calculate their body mass indices (BMI).

### 2.3 Source of yam and preparation of test foods

The yam including *Dioscorea rotundata* (white yam), *Dioscorea cayenensis* (yellow yam), and *Dioscorea alata* (water yam) were obtained from the open market in Techiman in the Brong‐Ahafo Region, Ghana. The tubers with defects were sorted out. The tubers were cooked using boiling, frying, and roasting. For frying and boiling, the yam tubers were peeled, washed, and sliced. Whereas the unpeeled sliced tubers were used for the roasting. Deep frying was done on the sliced yams for 35–40 min in vegetable oil after which the sliced yam was soft in texture and slightly brown in color. Boiling was done at 100°C for 35–45 min after which a soft texture was observed which indicated adequate cooking. Roasting was done using a local method at about 120°C for 40–45 min. Cooked food samples were cooled and served at room temperature.

### 2.4 Glycemic index determination

The glycemic index was determined as previously reported (Yeboah et al., 2019). After the anthropometric measurements, capillary blood was taken from each participant to assay for the fasting blood sugar (FBS). The Oral Glucose Tolerance Test (OGTT) was conducted as a control in this study. Subjects were each given a glucose solution prepared from 50 g glucose and 250 ml of bottled water. The stop watches were started when subjects begun drinking the glucose solution. The glucose solutions were taken within a period of 5 min. The time each participant began to drink the glucose solution was recorded. Fifteen (15) min after the start of consumption of the glucose solution, capillary blood was taken from each participant and assayed for glucose. Subsequently, blood samples were taken from all participants at the 30th, 45th, 60th, 90th, and 120th min as well and assayed for the glucose concentration in mmol/L. The same timing procedures were repeated for measuring the blood glucose concentrations of each participant after the test food samples (50 g equivalent of carbohydrate yam diet) were served. Total blood glucose levels were determined using the ACCU‐CHEK^®^ Perfoma glucometer (Roche Diagnostics). The blood glucose levels after consumption of the different yam diets/treatments were determined after a minimum of two (2) days allowed between the consumption of each yam product to ensure there was no residual effect of one treatment over the other. The same participants were used for all the test carried out. Glycemic response curves were plotted from the average blood glucose concentrations of all participants against time using the GraphPad Prism software version 5.00. The incremental area under the glucose response curves (IAUC) were calculated using the trapezoid rule as recommend (FAO/WHO, 1998). The area under the fasting baseline was ignored in the calculation. All GIs that were 2 standard deviations above or below the mean GI value for a given test were ignored as an outlier (Wolever et al., 1991). The glycemic index (GI) for the test food samples was calculated as:

$$\text{GI}\;(\%)=\left(\text{IAUC of sample}/\text{IAUC of glucose}\right)\times 100.$$

The average of the two measures for each subject was taken as the GI for that yam for the subject. The GI for each yam diet was finally calculated as the mean of the average of the GIs in ten subjects in the group. The GI of each yam for the subjects was compared between variety and treatment/cooking method.

### 2.5 Statistical analysis

The averages and standard deviations of all determined parameters were calculated using Microsoft Excel. A two‐way repeated measures ANOVA was carried out on data to compare between‐subjects effect of IAUC and GI as influenced by yam variety and treatment method at 5% significance level using the Statistical Package for Social Sciences (SPSS, IBM SPSS Statistics v20).

## 3 RESULTS AND DISCUSSION

### 3.1 Characteristics of test subjects

The test subjects were of average age, weight, and height of 22 ± 2 years, 62.21 ± 6.13 kg and 1.64 ± 0.08 m, respectively. They had mean body mass index (BMI) of 23.00 ± 1.74 kg/m^2^ and mean fasting blood sugar (FBS) of 4.45 ± 0.43 mmol/L.

### 3.2 Glycemic response curves

**FIGURE 1:**
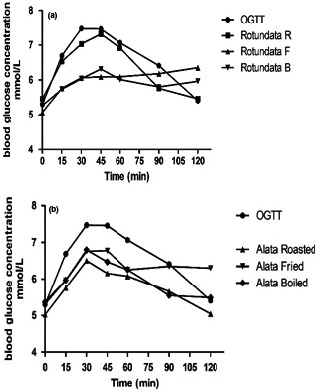
Glycemic response curves of roasted (R), fried (F) and boiled (B) yam diets: (a) *Dioscorea rotundata and* (b) *Dioscorea alata*. OGTT, Oral Glucose Tolerance Test

The glycemic response curves of glucose (reference food) and the test yam samples are as shown in Figure 1. The blood glucose concentration curves generally peaked after 30 min of consumption of test yam foods among the selected subjects. The highest peak (8.3 mmol/L) was observed in the fried yellow yam curve while fried and boiled white yam sample curves peaked the least at 6.1 mmol/L. The most flattened glycemic response curves were obtained in fried white yam samples. According to Anyakudo (2014), diets with the lowest glycemic indices had the most flattened glycemic response curves. However, the observation in this study was on the contrary since there was no significant difference between the glycemic indices of samples with flatter curves and peaked curves. The peak time (30 min) for the yam samples in this study was shorter than the 1 hr reported for both pounded and boiled yam but equal to that reported for *amala* (Jimoh et al., 2008). The 45‐min peak time also obtained in the study of Kouassi et al. (2009) for water‐cooked and oven‐cooked white and yellow yam varieties were higher than the findings in this study. A shorter peak time may indicate a higher tendency to quickly increase the blood glucose concentration after consumption hence foods with shorter peak times could be recommended for energy boosters for athletes.

### 3.3 Glycemic index of yam diet samples

Generally, the glycemic index of the yam diets ranged between 44.26 ± 12.67% in the boiled white yam diet to 75.18 ± 22.19% in the boiled yellow yam diet (Table 1).

**TABLE 1:** Incremental area under the curve (IAUC) and glycemic index (GI) of glucose and test yam diets in the study subjects

Food item	IAUC	GI (%)	GI class^a^
Glucose	155.76 ± 18.02	100	H
White yam (*D. rotundata*)			
Roasted	111.55 ± 26.12	50.62 ± 19.81	L
Fried	114.29 ± 19.01	59.13 ± 10.32	M
Boiled	71.45 ± 14.56	44.26 ± 12.67	L
Yellow yam (*D. cayensis*)			
Roasted	138.15 ± 20.86	70.62 ± 20.80	H
Fried	113.07 ± 15.42	65.08 ± 18.52	M
Boiled	126.53 ± 12.84	75.18 ± 22.19	H
Water yam (*D. alata*)			
Roasted	97.87 ± 13.87	54.04 ± 13.25	L
Fried	123.19 ± 26.53	69.16 ± 24.33	M
Boiled	80.19 ± 19.97	50.12 ± 18.91	L

^a^ GI Low (L): ≤55; Medium (M): 56–69; High (H): ≥70.

However, boiled and roasted yellow yam diets could be classified as high glycemic index foods since they both recorded GI of 70% and above. Whereas all fried yam diet samples could be classified as medium class glycemic index foods (Eli‐Cophie et al., 2017; Itam et al., 2012; Yeboah et al., 2019). The statistical comparison of GI of each yam for the subjects between variety and treatment/cooking method by means of a two‐way repeated measures ANOVA, however, indicated that GI varied insignificantly (*p* > .05) among the yam types and processing methods studied (Table 2).

**TABLE 2:** Test of Between‐Subjects effect of incremental area under the curves (IAUC) and glycaemic indices (GI) as influenced by yam variety and treatment

Yam diet	Significance (*p*‐value)*	
	IAUC	GI
Variety	0.158	0.449
Treatment	0.207	0.862
Variety × Treatment	0.475	0.945

* *p* > .05 implies no significant difference.

High glycemic index foods have the tendency to rapidly increase the blood glucose concentration after consumption and are therefore not recommendable to diabetic individuals (Brand‐Miller et al., 2003). Lower glycemic index foods on the other hand could be employed in the diabetic diets (Itam et al. (2012). Therefore, boiled white yam diet which recorded lower GI could be employed in the management of diabetes.

Variety and cooking can cause variations in the glycemic index yam as reported by Kouassi et al. (2009). The structure of carbohydrates differ from variety to variety and are also altered by the food processing methods such as boiling, roasting, baking, steaming, and pounding (Jimoh et al., 2008; Allen et al., 2012; Anyakudo, 2014; Itam et al., 2012; Kouassi et al., 2009).

The glycemic index of the roasted yam diets ranged from 50.62 ± 19.81% in the white yam to 70.62 ± 20.80% in the yellow yam variety (Table 1). The glycemic indices of roasted white yam in the present study were lower than the 64.00 ± 10.17% as reported by Anyakudo (2014). Roasting is reported to cause the breakdown and weakening of the structure of starch granules which subsequently leads to increased glycemic index in roasted foods (Bahado‐Singh et al., 2006) than in fried and boiled foods. The findings, however, were contrary to this earlier report since the roasted yam diets generally had lower glycemic indices compared to the fried yam diets.

The glycemic index (GI) of the fried yam diets ranged from 59.13 ± 10.32% in white yam to 69.16 ± 24.33% in water yam variety (Table 1). The glycemic indices of the fried foods were generally higher than the GIs of the roasted and boiled yam diets. The 59.13% GI recorded for white yam was also higher than the 24.50% reported by Anyakudo (2014). These differences in the GI of white yam could be attributed to the differences in the soils upon which yams were cultivated, length of storage of yam and methods of frying.

The glycemic indices of the boiled yam diets ranged from 44.26 ± 12.67% in white yam to 75.18 ± 22.19% in yellow yam. The glycemic index of boiled white yam diets was lower than those previously reported by others (Jimoh et al., 2008; Anyakudo, 2014). Boiling, followed by cooling lead to the formation of resistant starches and lower glycemic indices in potatoes, unripe bananas, and unripe plantain (Bahado‐Singh et al., 2006; Buyken & Kroke, 2005; Han et al., 2006; Nilsson et al., 2008). This could account for the relatively lower glycemic indices of boiled white yam and water yam diets recorded in the present study. Boiling could therefore be employed in the cooking of yam for consumption by diabetics since it has led to the formation of resistant starches and subsequent decreased glycemic index in foods.

## 4 CONCLUSION

White yam (*Dioscorea rotundata*) diets relatively had lower glycemic indices compared to yellow yam (*Dioscorea cayenensis*) and water yam (*Dioscorea alata*) diets. Fried yellow yam diets recorded relatively higher glycemic indices. The results showed that White‐yam‐boiled had the lowest GI (44.26%) followed in the increasing order by Water‐yam‐boiled (50.12%), White‐yam‐roasted (50.62%), Water‐yam‐roasted (54.04%), White‐yam‐fried (59.13%), Yellow‐yam‐fried (65.08%), Water‐yam‐fried (69.16%), Yellow‐yam‐roasted (70.62%) and Yellow‐yam‐boiled (75.18%).

Variation in glycemic index of the yam diets as influenced by variety and cooking method was not significant. However, boiling was found to give generally lower glycemic indices and could therefore be applied in the preparation of diets with lower GI diets for diabetics.

## CONFLICT OF INTEREST

The authors declare that they have no conflicts of interest.

## Data Availability

The data that support the findings of this study are available from the corresponding author upon reasonable request.
